# Seroprevalence of HBV, HCV & HIV Co-Infection and Risk Factors Analysis in Tripoli-Libya

**DOI:** 10.1371/journal.pone.0098793

**Published:** 2014-06-17

**Authors:** Mohamed A. Daw, Amira Shabash, Abdallah El-Bouzedi, Aghnya A. Dau, in association with the Libyan Study Group of Hepatitis & HIV

**Affiliations:** 1 Department of Medical Microbiology, Faculty of Medicine, Tripoli, Libya; 2 Department of Laboratory Medicine, Faculty of Biotechnology, Tripoli, Libya; 3 Department of Surgery, Tripoli Medical Centre, Faculty of Medicine, Tripoli, Libya; University of Cincinnati College of Medicine, United States of America

## Abstract

**Background:**

In 1998 Libya experienced a major outbreak of multiple blood borne viral hepatitis and HIV infections. Since then, no studies have been done on the epidemic features and risk factors of HBV, HCV, HIV and co-infection among the general population.

**Methods:**

A prospective study was carried out using a multi-centre clustering method to collect samples from the general population. The participants were interviewed, and relevant information was collected, including socio-demographic, ethnic, and geographic variables. This information was correlated with the risk factors involved in the transmission of HBV, HCV and HIV. Blood samples were collected and the sera were tested for HBsAg, anti-HCV and anti-HIV using enzyme immunoassay.

**Results:**

A total of 9,170 participants from the nine districts of Tripoli were enrolled. The average prevalence of HBsAg was 3.7%, anti-HCV 0.9%, anti-HIV 0.15% and co-infection 0.02%. The prevalence varied from one district to another. HBV was more prevalent among those aged over 50 years and was associated with family history. Anti-HCV and anti-HIV were more prevalent among those aged 20–40 years. Intravenous drug use and blood transfusion were the main risk factors for HCV and HIV infection.

**Conclusion:**

HBV, HCV, HIV and co-infection are relatively common in Libya. High prevalence was associated with geographic, ethnic and socioeconomic variability within the community. HCV and HIV infections among the younger age groups are becoming an alarming issue. Regulations and health care education need to be implemented and longer term follow-up should be planned.

## Introduction

Hepatitis B (HBV), hepatitis C (HCV) and human immune deficiency (HIV) viruses are among the most commonly known viruses worldwide, and they have gained more attention than many other pathogens. Their impacts go beyond the infected individuals to affect national economies and even modulate certain societal and personal behaviours [1]. Despite their biological differences, these viruses share common routes of transmission and similar risk factors [2]. Worldwide, HBV accounts for about 370 million chronic infections, HCV for an estimated 130 million, and HIV for about 40 million. About 2–4 million people infected with HIV have chronic HBV co-infection and 4–5 million have HCV co-infection [3]. The prevalence rates vary greatly from one region to another and over time. Hence, surveillance studies are needed to monitor the prevalence patterns of these viruses and to implement appropriate preventive measures.

In Africa, HBV, HCV and HIV infections are considered to be endemic, but their rates are highly variable among the African countries. HBV and HCV prevalence rates range from 3–20% and 1–26%, respectively. Furthermore, over 63% of those infected with HIV worldwide reside in Africa, and 2.7 million new HIV infections were reported in Sub-Saharan Africa alone in 2008 [4], [5].

Up-to-date information on the epidemiology and burden of disease attributable to HBV, HCV and HIV is essential for the development of appropriate national policies in any country. In Libya, different studies were carried out on the prevalence of HBV and HCV infections [6], [7]. A large cross-sectional study carried out between 2005 and 2006 showed that the prevalence of HBV ranged from 1.4% to 6.6%, and for HCV from 0.6% to 2.2% [8]. The common risk factors associated with these infections were blood transfusion and intravenous drug use (IVDU). However, none of these studies lend themselves to analysis of the prevalence of HIV infection and its association with HBV and HCV infections. Lack of adequate data on HIV infection among the Libyan population leaves the matter open to speculation.

HIV infection is a growing pandemic in Africa, and data on the prevalence of HBV and HCV among HIV infected individuals are scanty. In developing countries, liver disease due to chronic HBV and/or HCV has become a growing problem, particularly in those infected with HIV [9]. Therefore, it is important to document HIV co-infections in regions with high hepatitis chronicity and HIV infection rates. Indeed, HIV accelerates the progression of chronic liver diseases related to HBV and HCV. Furthermore, most HIV patients are usually co-infected with viral hepatitis, which means that liver diseases will likely emerge as significant causes of morbidity and mortality among HIV infected individuals in Africa, similar to the trend worldwide [10].

In 2013 Daw *et al*. [11]. constructed a mathematical model to trace HIV/AIDS epidemics among Libyan children. The study showed that the prevalence of HIV was 0.015% in 2012 and estimated that it will increase about three folds by 2022 [11]. An increase in HIV infections among the Libyan population will have major social and health consequences. Efforts should be undertaken to contain the consequences of HIV infections, particularly those associated with co-infection with HBV and/or HCV.

In Libya, bloodborne hepatitis and HIV attracted major international attention in the context of the Bulgarian nurses saga and the controversy it generated [1], [12], [13]. More than 440 Libyan children were infected with HIV at the same hospital within a short period of time, and 47% of them were co-infected with HCV and 37% with HBV [14], [15]. This outbreak was considered the largest of its kind. However, there have been no studies on the epidemic features and risk factors of co-infection among the Libyan population. Hence, it is necessary to investigate the prevalence of co-infection of HBV, HCV and HIV among Libyans in order to plan and implement the necessary remedial programs. The objectives of this study were to determine the prevalence rates of HBV, HCV and HIV in Tripoli, Libya, and to analyse the risk factors associated with co-infection with these viruses.

## Materials and Methods

### Study population and data collection

Tripoli, the capital and the largest city in Libya, had a population of 1,879,000 in the national census of 2004–2005. We solicited the participation of 10752 adults aged from 16 to 72 years from the nine districts of Tripoli. A total of 9170 residents agreed to participate and filled in the questionnaire, giving a response rate of 85.3% (Table 1). The participants completed the questionnaires, and those of a younger age or in need of assistance were helped by their parents or those accompanying them in the presence of physicians and social assistants, who recorded the needed information and coded the questionnaire. The questionnaire asks about age, occupation, medical history with emphases on blood transfusion, surgical exposure, haemodialysis, as well as skin piercing, IVDU and sexual behaviour.

**Table 1:** Total number of samples collected from nine Districts of Tripoli-Libya 2011.

District	Number	Percentage(%) Ratio*
Old town	790	10.7 1∶70
Souk Aljoma	980	10.8 1∶80
Tajora	970	10.7 1∶100
Al-Hadba	1200	13.3 1∶100
Bosaleam	1850	20.4 1∶90
Gaser Bengahser	780	8.6 1∶80
Quatashal	880	9.7 1∶100
Gorjy	870	9.6 1∶100
Janzour	850	9.3 1∶80
Total Sample	9050	100 1∶80

*Ratio of tested individuals for hepatitis B, C, HIV viruses to the total population of Tripoli

### Laboratory testing

A blood sample of 5–10 ml was collected from each participant using a sterile plain vacutainer, and the serum was separated by centrifugation and placed in sterile serum storage vials. Needles were destroyed using a needle destroyer and then discarded in a sharps box. The samples were analysed in Tripoli Medical Reference Laboratory. All samples were tested for HBsAg and anti-HCV markers by using a third-generation enzyme immunoassay (Axsym; HCV EIA 3.0; Abbott Laboratories, Abbott Park, Illinois, and HBsAg, Axsym, Switzerland) as previously described [17]. Samples were considered reactive according to the manufacturer's specifications. Hepatitis B infection was defined as the presence of hepatitis B surface antigen in the serum detected by ELISA, while hepatitis C and HIV infections were defined as the presence of anti-hepatitis C and HIV antibodies (IgG) in the serum detected by ELISA. All positive results were confirmed being positive as previously described [17].

### Data analysis

The data were coded and entered in an excel sheet. The data-base was cleaned and verified. The data were analysed by using Microsoft Excel 2007, and statistical tests were done using Minitab version 15 and SPSS version 16. A chi-square test was used to determine the difference in risk factors between sero-positive and sero-negative viral status. Odds ratios (OR) and 95% confidence intervals (CI) in univariate analysis were calculated using seronegative persons as reference. Multiple logistic regression was used for multivariate analysis to determine the independent risk factors for exclusively HBsAg(+), anti-HCV(+) or anti-HIV(+).

### Ethical approval

The study was approved by the Libyan National Ethical Committee (Approval No. LY NS; HV-CBHIV-299761). It was conducted in accordance with the Helsinki Declaration [16] and under the supervision of the Faculty of Medicine, Tripoli, Libya. All participants signed an informed consent form witnessed by the local health office before collection of data and blood samples. The questionnaire used to collect demographic and epidemiological data was anonymous and linked to the blood sample tube only by a code.

## Results

Serum samples were collected from 9170 individuals enrolled from the nine districts of Tripoli. The male-to-female (M∶F) ratio was 4.4∶1.0 (7500 males, 81.8%; 1670 females, 18.2%). The ages of the participants ranged from 16 to 72 years, with a mean of 34±16.30 years. The serum samples were analysed for HBsAg, anti-HCV and anti-HIV antibodies. Of the 9170 samples, 438 (4.9%) were singly positive for HBsAg, anti-HCV or anti-HIV.

**Figure 1:**
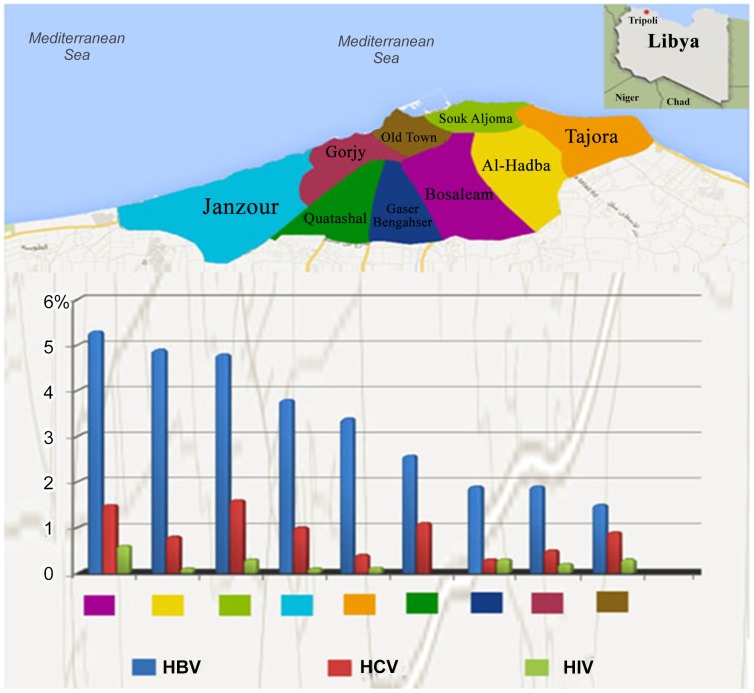
Map of Tripoli showing the Location of 9 studied districts and Sero-prevalence of HBV, HCV and HIV among each district, Tripoli-Libya 2011.

The prevalence of the three viruses varied from one district to another (Figure 1). The overall prevalence of HBV was 3.7% (95% CI 3.6%–3.8%). The highest prevalence rates of HBsAg were in Abu Saleem district (5.2%), Alhadba and Souk Aljuma (4.75% each). It was less prevalent in the Old City (1.5%) followed by Qasr Ben Gashir (1.9%). The prevalence of HBV was 1.4 times higher among males.

The overall prevalence of HCV was 0.9% (95% CI, 0.8%–1.1%). The prevalence was highest in Abu Saleem (1.5%) and Souq Aljuma (1.3%), and lowest in Qasr Ben Gasher (0.1%) and Tajura (0.2%). The prevalence was slightly higher in females (M∶F ratio 1.0∶1.1).However, It is important to highlight the fact that a portion of these individuals with confirmed antibodies may have spontaneously cleared though additional data is needed to assess this. The highest prevalence rates of HIV were in the Old city (0.4%), Abu Saleem (0.3%), Souk Aljuma (0.2%) and Gurji (0.2%). In the other districts it was 0.1% or less. The prevalence was higher among males (M∶F ratio 3.6∶1.0).

Co-infections among the studied population are presented in table 2. The highest rate was co-infection with HCV and HIV which was found to be 14(0.15%) (p<0.001) among the infected persons. This is higher that of HCV/HBV, HBV/HIV, and HBV/HCV/HIV co-infections which were 4(0.04%), 3(0.03%) and 2(0.02%) respectively. All those co-infected by HCV and HIV had a history of IVDU.

**Table 2:** Prevalence of HBV, HCV and HIV in Tripoli.

	No. positive (%)		Ratio	Total (%)
	Males	Female	M∶F	No. positive (%)
Prevalence of HBV infection	265 (78.4)	73(21.6)	(3.6∶1)	338 (3.7)
Prevalence of HCV infection	42 (48.8)	44(51.2)	(1;1.1)	86 (0.9)
Prevalence of HIV infection	11 (78.7)	3(21.4)	(3.7∶1)	14 (0.15)
Co-infection of HBV/HCV	3 (75.0)	1(25.0)	(3∶1)	4 (0.04)
Co-infection of HBV/HIV	2 (66.7)	1(33.3)	(2∶1)	3 (0.03)
Co-infection of HCV/HIV*	10 (83.3)	2(16.7)	(3.7∶1)	12 (0.13)
Co-infection of HBV/HCV/HIV	2 (100.0)	0(0)	(2∶0)	2 (0.02)

n = 7500 males and 1670 females. * Statistically significant at *P* = 0.001

**Figure 2:**
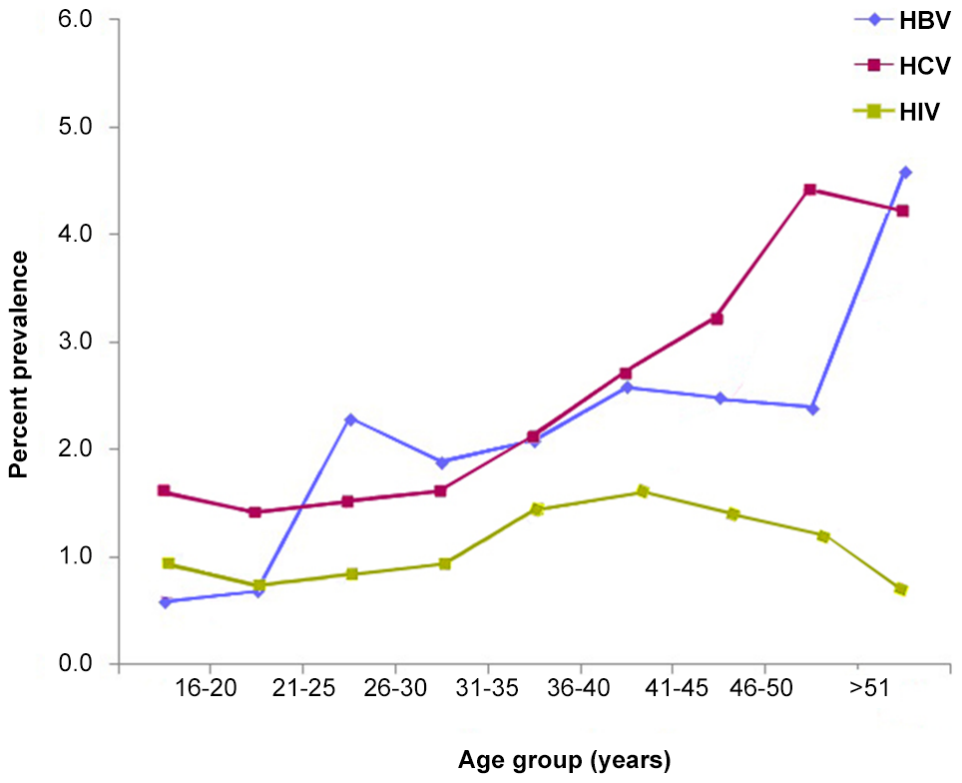
Prevalence of hepatitis B, C and HIV infections by age, Tripoli -Libya 2011.

The correlation of age with the prevalence of HBV, HCV and HIV is shown in figure 2. The age group most infected with HBV was 18–29 years old. The prevalence of HBsAg was lowest in the youngest age group (0.9%) while in the older groups it fluctuated between 2.0% and 2.9% without any evident temporal pattern. However, it rose sharply after the age of 50 years. The mean age of HBsAg-positive individuals was 36±16.8 years among females and 39±19.8 among males.

In contrast, the prevalence of anti-HCV increased with age, rising gradually after the age of 30 years: from 0.7%–0.9% in those ≤30 years old to 3.7% in those >30 years. The prevalence of anti-HCV was stable within the younger ages but increased steadily after the age of 30 years to reach a peak at the age of 50 years. The age group most infected with HCV was 33–40 years old. The prevalence of HIV showed two peaks, the higher one among those aged 26–30 years and the other in those aged 21–25 years. The mean age of anti-HIV-positive individuals was 31.7±18.4 years among females and 35.6±20.9 years among males.

Risk factor analysis was carried out on different factors associated with infection: gender, age, hospital admission, dental procedure, blood transfusion, IVDU, surgical exposure, haemodialysis, family history, and sexual behaviour. The analyses of risk factors and demographic characteristics are shown in table 3. According to univariate analysis, dental treatment, traditional exposures (such as tattooing and skin piercing) and gender were not significantly associated with HBV, HCV or HIV infection. IVDU, blood transfusion, hospital admission and haemodialysis were associated with increased risk of HCV infection (P<0.001). HIV infection was associated with IVDU (P<0.001). Risk factors for HBV infection were mainly direct contact with infected individuals and a family history of HBV infection.

**Table 3:** Association between HBV,HCV and HIV infections and certain influencing variables among Tripoli population.

	HCV			HBsAg			HIV		
	OR	95.0% C.I.		OR	95.0% C.I.		OR	95.0% C.I.	
		Lower	Upper		Lower	Upper		Lower	Upper
Age group									
16–20	0.53	0.34	1.12	1.12	0.67	1.24	0.41	0.29	1.01
21–25	0.70	0.43	1.02	2.89	1.95	3.95	0.60	0.35	0.95
26–30	0.67	0.45	1.02	2.91	2.21	3.9	0.59	0.39	0.91
31–35	1.23	1.02	1.85	3.02	2.12	4.11	1.04	1.01	1.62
36–40	1.56	1.22	2.43	3.55	2.30	4.97	1.37	1.01	2.06
41–45	1.25	1.44	2.87	3.3	2.25	4.88	1.07	1.02	2.08
46–50	2.16	1.15	3.82	3.12	1.95	4.94	2.04	1.01	3.02
>51	2.21	1.01	4.38	5.58	3.23	8.95	2.02	0.94	4.05
Males vs females	0.75	0.63	0.89	1.62	1.46	1.79	1.71	1.68	1.98
Blood transfusion	1.22	1.12	1.87	0	0	0	0	0	0
Intravenous drug use	6.31	2.15	17.15	0	0	0	5.65	1.75	17.06
Surgical exposure	1.21	1.05	1.43	0	0	0	0	0	0
Family HBV	1.12	1.1	1.90	1.65	1.34	2.54	0	0	0
Haemodialysis	3.89	1.81	10.40	0	0	0	0	0	0
Contact with HBV				1.51	1.12	2.13	0	0	0

## Discussion

Sero-epidemiological studies on HBV, HCV and HIV are an important step in formulating preventive strategies. The prevalence rates of these infections vary according to the risk factors involved, socioeconomic status, and initial burden of infectious markers in the community, which vary from one country to another and even between different regions within the same country. Such data are rarely available in African countries [18]. In Libya, data on co-infection of HIV, HBV and HCV are lacking, and the influence of geographic variability, ethnicity and socioeconomic factors on these infections has not been studied. In recent years Libya has attracted worldwide attention because many Africans transit through it to enter Southern Europe illegally, with the possibility of transmitting infection in transit, or at the destination. The uprising and armed conflict in Libya in 2011 diminished control on the borders, and moreover, it had negative effects on socioeconomic status and health care services in Libya [19]. Hence, studies on the sero-prevalence of HBV, HCV and co-infection with HIV and the associated risk factors are needed.

HBV and HCV are well documented in Libya, but no data are available on the prevalence of co-infection with HIV [8], [20]. Here, we determined the prevalence of HBV, HCV and HIV and their co-infection rates among the Libyan population in Tripoli. Studying the prevalence of these infections will provide insights into their effects on the Libyan community and the effect of co-infection on the progress of the diseases. Such information is needed to institute more effective preventive measures.

The overall prevalence of HBV in Tripoli was 3.7%. The highest prevalence was in Abu Saleem (5%), which is higher than the reported 2.5% prevalence among the general population (8). The lowest was in the Old city (1.5%). The prevalence of HCV was 0.9%, which is lower than reported in Libya in general (1.3%) and in neighbouring countries: Algeria (2.5%), Tunisia (1.3%), and Morocco (1.6%) [8], [21], [22], [23].The prevalence of HCV varied from one district to another: the highest was in Abu Saleem (1.5%) and lowest in Qasr Ben Gashir (0.1%). The high prevalence of HBV and HCV in Abu Saleem and Alhadba could be related to socio-economic conditions, as residents of these districts have lower incomes and higher rates of unemployment, and they house large numbers of young people with drug addiction. Such variation has also been reported in other Mediterranean countries. In Italy, Greece, France and Spain, the prevalence of these viruses varies greatly from one region to another within the same country [24], [25], [26].

We observed an overall prevalence of HIV in the capital of Libya of 0.15%, which categorizes Libya as having a very low prevalence. The highest HIV prevalence was found in the Old City (0.4%), where most of the inhabitants are immigrants, mainly from Sub-Saharan Africa. The data are similar to most other studies in North African and Middle-Eastern countries [27]. The low levels of HIV prevalence in these countries could be attributed to the conservative nature of these societies. But there is no reason for complacency because HIV is now spreading at alarming rates among North African countries and the picture could become substantially different in the near future. Further studies are needed, and efforts should be made to combat the spread of HIV and associated co-infections [11], [27], [28].

We found that the prevalence rate of HBV, HCV and HIV varied with age. HBsAg was more prevalent among males in all age groups. By contrast, the prevalence of anti-HCV was higher among females and increased with age, rising gradually after the age of 30 years. Likewise, anti-HIV was most prevalent in the age group 26–30 years, followed by the 21–25 year age group. The mean age of anti-HIV-positive was 31.7±18.4 years in females and 35.6±20.9 years in males. These results are in agreement with those reported in Egypt and Morocco [29], [30].

The main risk factors of HBV in this study were family history and direct contact with HBV-positive individuals. This pattern is unlike that in Sub-Saharan Africa, where HBV is commonly transmitted during childhood between siblings long before infection with HIV and HCV [31]. In Libya, HCV was mainly associated with blood transfusion and certain other health care facilities, such as haemodialysis units. Furthermore, both HCV and HIV were prominent among IVDUs. This is in agreement with studies reported from other African countries [32], [33]. It is important to identify the high-risk groups due to their potential roles in spreading infections.

Studying patterns of co-infection with HBV, HCV and HIV is of great importance, particularly in the context of controlling morbidity and mortality caused by liver disease. Little is known about the prevalence HIV and hepatitis co-infection among Libyans. Our study was devoted to this purpose. HIV co-infection is known to influence the clinical outcome of patients with HBV or HCV infection, accelerating the progression to advanced liver disease among them [34]. The prevalence of HBV, HCV and HIV co-infection in our study is lower than that in other North and Sub-Saharan African countries [35], [36]. This may be due to the higher socioeconomic status of the Libyan population. However, additional information is needed, particularly on the high risk groups, such those incarcerated and IVDUs [37].

Independent predisposing factors for HBV and HCV co-infection were found within our study. An association between HBV infection and HCV infection indicates shared transmission routes, and particularly a history of blood transfusion. Such co-infection was found to be very limited among the population studied: only 0.04% of the participants were found to be co-infected with HBV and HCV, which is not markedly different from the rate for HIV and HBV co-infection. This rate is very low compared to those observed in Tunisia (5%) and Egypt (22.5%) [38], [39]. Our study suggests that people infected with HIV and HCV do not have greater exposure or susceptibility to HBV than the general population. This may be related to the low prevalence of HBV and HCV among Libyans and the compulsory use of HBV vaccination in Libya.

HIV modifies the natural history of HCV infection, with clear evidence of a higher viral load and accelerated liver disease progression in persons with HIV and/or HCV co-infection [40]. Intriguingly, HCV/HIV co-infection was the most predominant co-infection among Libyans, at a rate 0.14% comparable to HBV/HCV and BHV/HIV co-infection (0.03%).The major determinant of HCV and HIV co-infection was IVDU: approximately all of those co-infected by HCV and HIV were IVDUs, and this type of co-infection was more prevalent in poor communities. This is in agreement with a case control study recently carried out in Vietnam, where 98.5% of HIV-infected IVDUs were co-infected with HCV [41]. Longer-term follow-up and further studies are required to examine the effect of co-infection on the clinical outcome among Libyans co-infected with HBV, HCV and HIV[42].

We observed gender differences in infection rates despite that most of those participated in the study were males (4;1). This may reflect the conservative nature of Libyan society and highlights the need to improve sampling techniques particularly among women sectors in the upcoming surveys. HBV was more prevalent in males but HCV was more prevalent among females. The prevalence rates of HIV and of HBV/HCV/HIV co-infections were higher among males. Similar observations have been reported from North African and other Arab countries [1], [28], [32]. It is unclear whether this gender distribution exists in the general population. Our results are in agreement with other studies carried out in Morocco, Algeria, Tunisia and Egypt [43], [44], [45], [46]. However, they are at odds with findings in other studies from national population based surveys in 19 sub-Saharan African countries, which showed the predominance of females in infected groups, where the lowest female to male ratio reported was 2∶1 [47], [48]. This may suggest the geo-epidemiological variation of HBV, HCV and HIV in Africa. Hence, further studies are needed to clarify this variation. Our study has certain limitations that should be considered. First, HCV infection was based on detection by antibodies rather than detection of HCV RNA. Second, it did not include assessment of liver function. Third, the study was conducted in only one city, and therefore the risk factor analysis cannot be directly generalised to the entire population.

## Conclusion

This study gives clear insights into the epidemiology of these three viruses among Libyans, particularly the epidemiological status of HIV and co-infection, which has hardly been explored in previous studies[6], [7], [8]. Our study highlights the risks that Libyans may face and provides data that would be useful for designing measures to limit HBV, HCV and HIV infections and co-infections. Further to identifying the risk groups identified such as poor and marginalized communities. This emphasises the need to improve screening among groups less reflected in this survey (e.g. women) and introducing additional testing such as HCV RNA for such population.Clear national policies should be established in that context. They should include clear economic and health care strategies to improve the quality of living conditions, education and easy access to health care facilities, and to combat intravenous drug use [49].
